# Successful transarterial embolization of a neonatal tongue vascular malformation presenting with life-threatening airway compromise: a case report

**DOI:** 10.1186/s42155-026-00728-1

**Published:** 2026-07-11

**Authors:** Nisha Govindani, Shivaji Pole, Devidas Dahiphale, Rutuja Ukride, Abhijeet Nagapurkar, Karanvir Chhabra, Priyanka Jaybhaye

**Affiliations:** 1 Department of Radiology, MGM Medical College and MGM School of Biomedical Sciences, Chhatrapati Sambhajinagar, India; 2 Department of Radiology, MGM Medical College and Hospital, Chhatrapati Sambhajinagar, India; 3 Department of Radiology, MGM School of Biomedical Sciences, Chhatrapati Sambhajinagar, India; 4 Department of Radiology, R.K. Damani Medical College and Dr. Hedgewar Hospital, Chhatrapati Sambhajinagar, India

**Keywords:** Neonatal vascular malformation, Tongue vascular lesion, Lingual artery embolization, Digital subtraction angiography, Endovascular embolization, Pediatric interventional radiology

## Abstract

**Background:**

Vascular malformations of the tongue are rare congenital vascular anomalies that may present with bleeding, feeding difficulty, and life-threatening airway compromise in neonates. Prompt diagnosis and early intervention are essential because of the limited airway reserve in this age group. Magnetic resonance imaging (MRI) and digital subtraction angiography (DSA) play important roles in lesion characterization and treatment planning.

**Case presentation:**

We report the case of a 1-month-20-day-old infant weighing 3 kg who presented with progressive tongue swelling, intermittent bleeding, and respiratory distress requiring endotracheal intubation for airway stabilization. MRI demonstrated a vascular lesion involving the anterior tongue with multiple internal flow voids and homogeneous post-contrast enhancement. DSA revealed a hypervascular lesion supplied predominantly by branches of the lingual artery arising from the external carotid artery.

**Intervention and outcome:**

Superselective transarterial embolization was successfully performed using a microcatheter system and 250 μm polyvinyl alcohol (PVA) particles. Post-embolization angiography demonstrated significant reduction in vascular blush with successful devascularization of the lesion. Clinically, the infant showed cessation of bleeding, improvement in airway patency, and no immediate procedural complications.

**Conclusion:**

Transarterial embolization represents a safe and effective minimally invasive treatment option for neonatal tongue vascular malformations presenting with airway compromise. Careful technique and specialized neonatal endovascular expertise are essential to achieve favorable clinical outcomes while minimizing procedural complications.

## Introduction

Vascular malformations of the tongue are rare congenital lesions that may present with bleeding, feeding difficulty, and, in severe cases, life-threatening airway compromise in neonates. Due to the limited airway reserve and small anatomical dimensions in this age group, even modest increases in lesion size can result in rapid clinical deterioration, necessitating urgent evaluation and intervention [1, 2].

Imaging plays a pivotal role in diagnosis and treatment planning. Magnetic resonance imaging (MRI) provides detailed characterization of lesion extent and tissue composition, while digital subtraction angiography (DSA) remains the gold standard for identifying feeding vessels and facilitating endovascular management [3, 4].

Although transarterial embolization has emerged as an effective minimally invasive treatment modality for vascular malformations, its application in neonates remains technically challenging due to small vessel caliber, increased susceptibility to vasospasm, and a higher risk of procedure-related complications [4, 5]. Furthermore, detailed technical descriptions of embolization strategies in this population remain limited in the literature.

Herein, we report a case of neonatal tongue vascular malformation presenting with life-threatening airway compromise, successfully managed with superselective transarterial embolization, highlighting key technical considerations and clinical outcomes.

## Case presentation

A 1-month-20-day-old infant weighing approximately 3 kg presented with progressive swelling of the anterior tongue associated with intermittent bleeding, fever, feeding difficulty, and respiratory distress. The swelling progressively increased in size, resulting in significant airway compromise requiring endotracheal intubation for airway protection.

Clinical examination revealed diffuse enlargement of the anterior two-thirds of the tongue with prominent vascularity and mild surface irregularity. Focal areas of recent surface bleeding were noted. Significant tongue enlargement contributed to mechanical airway obstruction and feeding difficulty. No associated facial swelling, cervical masses, or lymphadenopathy were identified.

**Fig. 1:**
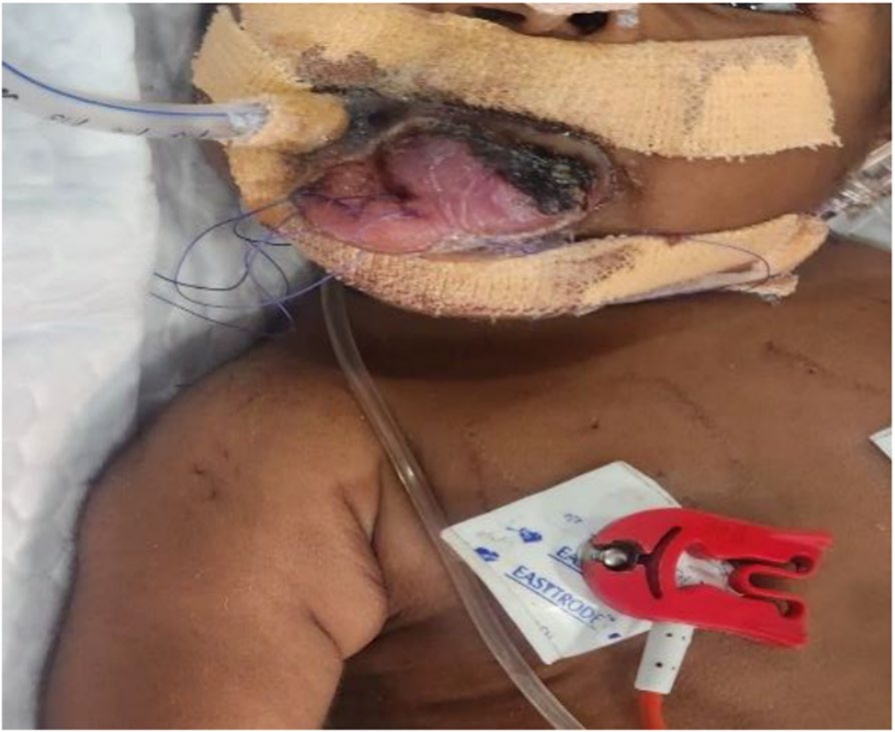
Pre-procedural clinical image demonstrating diffuse enlargement of the anterior portion of the tongue with a vascular-appearing lesion

Due to worsening respiratory distress and concern for airway obstruction, urgent imaging evaluation and multidisciplinary management were undertaken (Fig. 1).

## Diagnostic assessment

### MRI findings

Magnetic resonance imaging revealed an altered signal intensity lesion measuring approximately 2.6 × 3.2 × 1.9 cm (anteroposterior × transverse × craniocaudal) involving the anterior two-thirds of the tongue. The lesion appeared isointense on T1-weighted images and hyperintense on T2-weighted and STIR sequences with multiple internal flow voids and homogeneous post-contrast enhancement.

The lesion involved the bilateral genioglossus muscles, left geniohyoid muscle, and intrinsic longitudinal muscles of the tongue. These imaging features were suggestive of a vascular malformation (Table 1).

**Table 1:** Clinical and imaging summary

Parameter	Findings
Age	1 month 20 days
Weight	3 kg
Clinical presentation	Tongue swelling, bleeding, feeding difficulty, airway compromise
MRI findings	Hyperintense vascular lesion with internal flow voids
DSA findings	Hypervascular blush supplied by lingual artery
Final diagnosis	Tongue vascular malformation

### Differential diagnosis

**Fig. 2:**
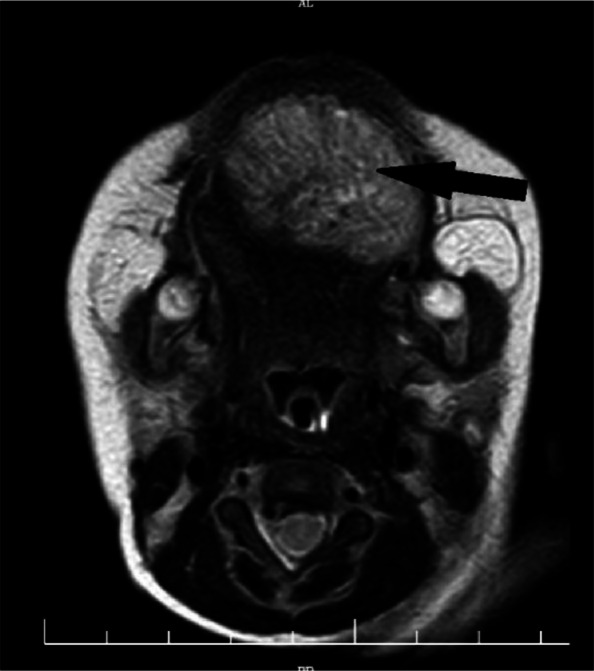
Axial T2-weighted MRI demonstrating a hyperintense lesion involving the anterior two-thirds of the tongue with internal flow voids (arrow), suggestive of a vascular malformation

Differential diagnostic considerations included infantile hemangioma, venous malformation, arteriovenous malformation, and lymphatic malformation. Based on the MRI characteristics and angiographic findings, a vascular malformation supplied predominantly by branches of the lingual artery was considered the most likely diagnosis (Fig. 2).

### Digital subtraction angiography

To further delineate the vascular supply of the lesion and facilitate therapeutic intervention, digital subtraction angiography (DSA) was performed.

Selective angiography of the external carotid artery demonstrated a well-defined hypervascular blush localized to the anterior portion of the tongue, corresponding to the lesion identified on MRI. The lesion was supplied predominantly by branches of the lingual artery arising from the external carotid artery, with multiple small tortuous arterial feeders contributing to the vascularity.

Superselective catheterization of the lingual artery was subsequently performed using a Progreat microcatheter and Neuro guidewire, allowing detailed visualization of the feeding vessels. No significant arterial supply from the facial or ascending pharyngeal arteries was identified. Additionally, no evidence of high-flow arteriovenous shunting or early venous drainage was observed.

**Fig. 3:**
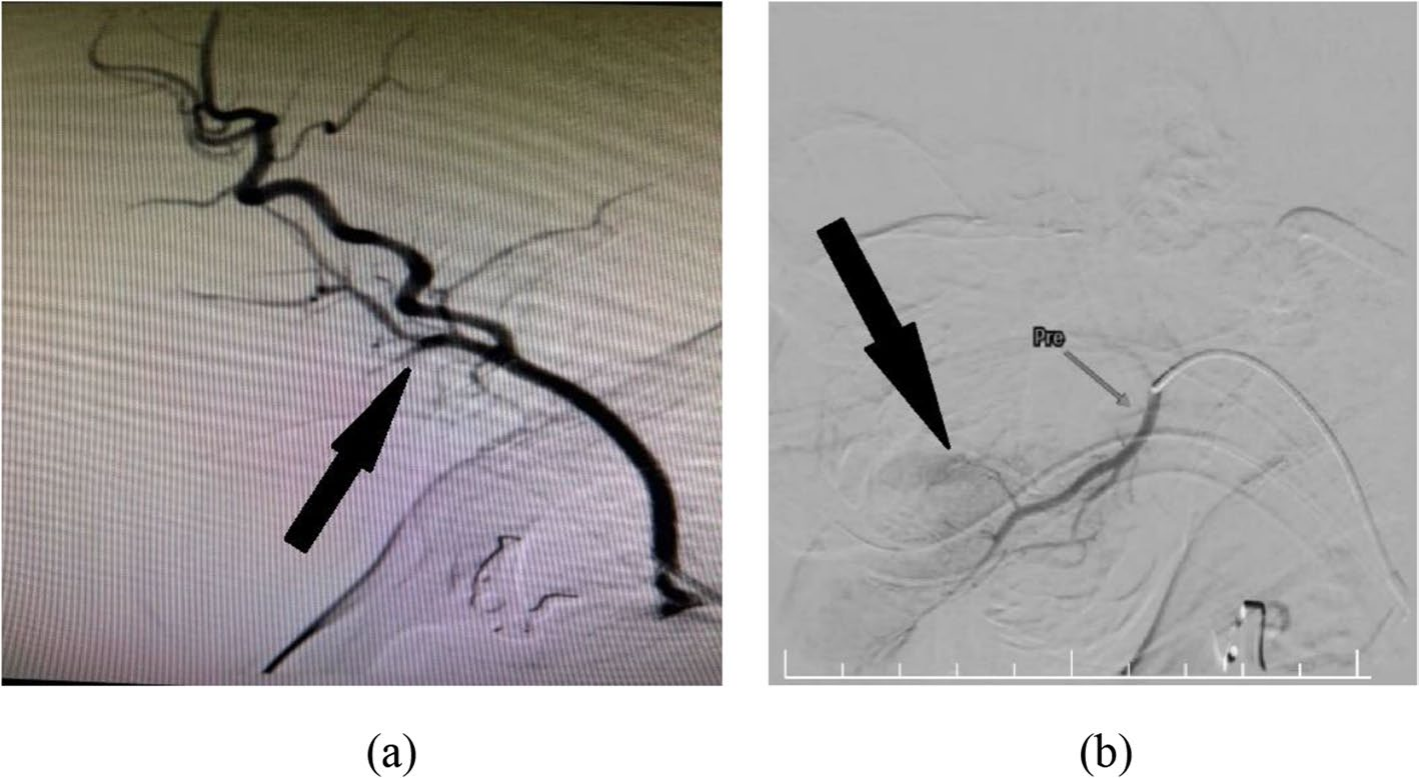
**a** Digital subtraction angiography demonstrating a hypervascular blush (arrow) within the anterior tongue lesion supplied by branches of the lingual artery. **b** Selective lingual artery injection demonstrating feeding arterial branches supplying the lesion (arrow)

These findings confirmed that the lesion was primarily supplied by branches of the lingual artery, making it suitable for selective transarterial embolization (Fig. 3).

### Therapeutic intervention

The procedure was performed under strict aseptic precautions. Vascular access was obtained via the right common femoral artery using a 4-French introducer sheath. A hydrophilic diagnostic catheter was advanced into the external carotid artery under fluoroscopic guidance.

Systemic heparin was administered at a dose of 100 IU/kg during the procedure. Continuous catheter flushing was maintained using heparinized saline (1 U/mL) to minimize catheter-related thrombosis.

Local anesthesia was administered using 2% lidocaine within safe neonatal dosage limits.

A Progreat microcatheter with a Neuro guidewire was used to achieve superselective catheterization of the feeding branch of the lingual artery. Careful catheter manipulation was performed to minimize vasospasm, and no vasodilators such as Nitroglycerin or verapamil were required during the procedure.

Embolization was performed using 250 μm polyvinyl alcohol (PVA) particles diluted with contrast in a 50:50 ratio. The embolic material was injected slowly under continuous fluoroscopic monitoring.

**Fig. 4:**
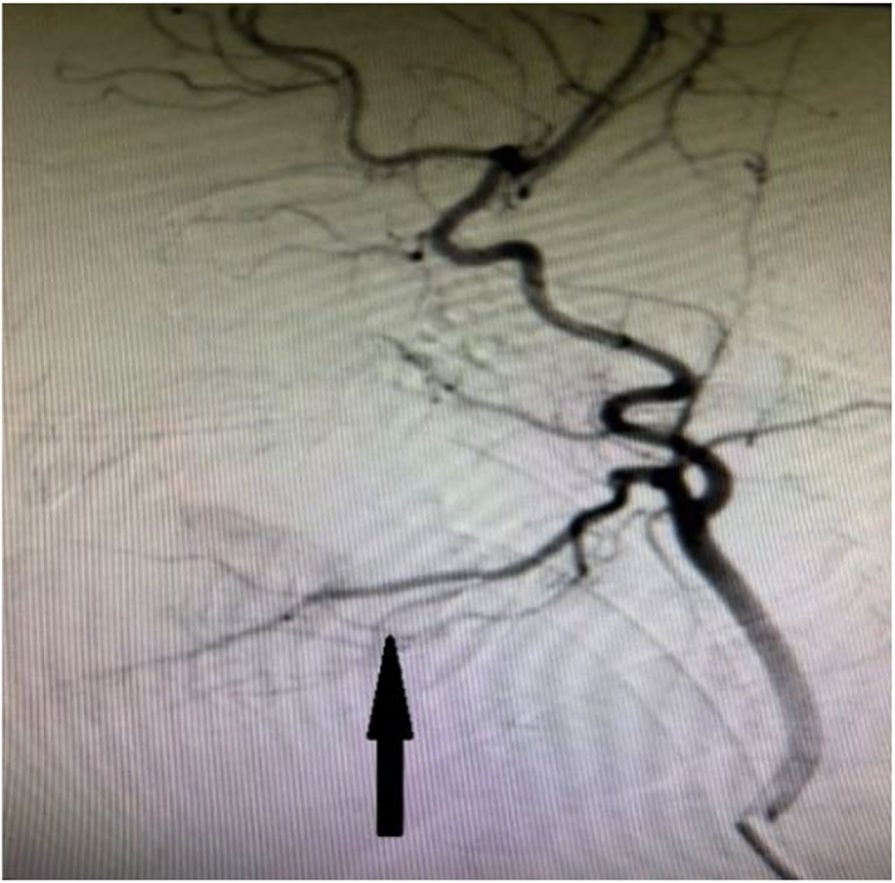
Post-embolization angiography demonstrating significant reduction in vascular blush (arrow) and successful devascularization of the tongue vascular malformation following selective Transarterial embolization

The endpoint of embolization was angiographic stasis with significant reduction in vascular blush. Post-embolization angiography demonstrated marked devascularization of the lesion (Fig. 4).

### Follow-up and outcomes

The patient was closely monitored in the post-procedural period. Post-procedural management included administration of antibiotics, analgesics, and anti-inflammatory medications. Steroids were not administered.

Gradual reduction in tongue swelling was observed with improvement in airway patency and feeding ability. The airway was stabilized successfully, allowing removal of airway support.

**Fig. 5:**
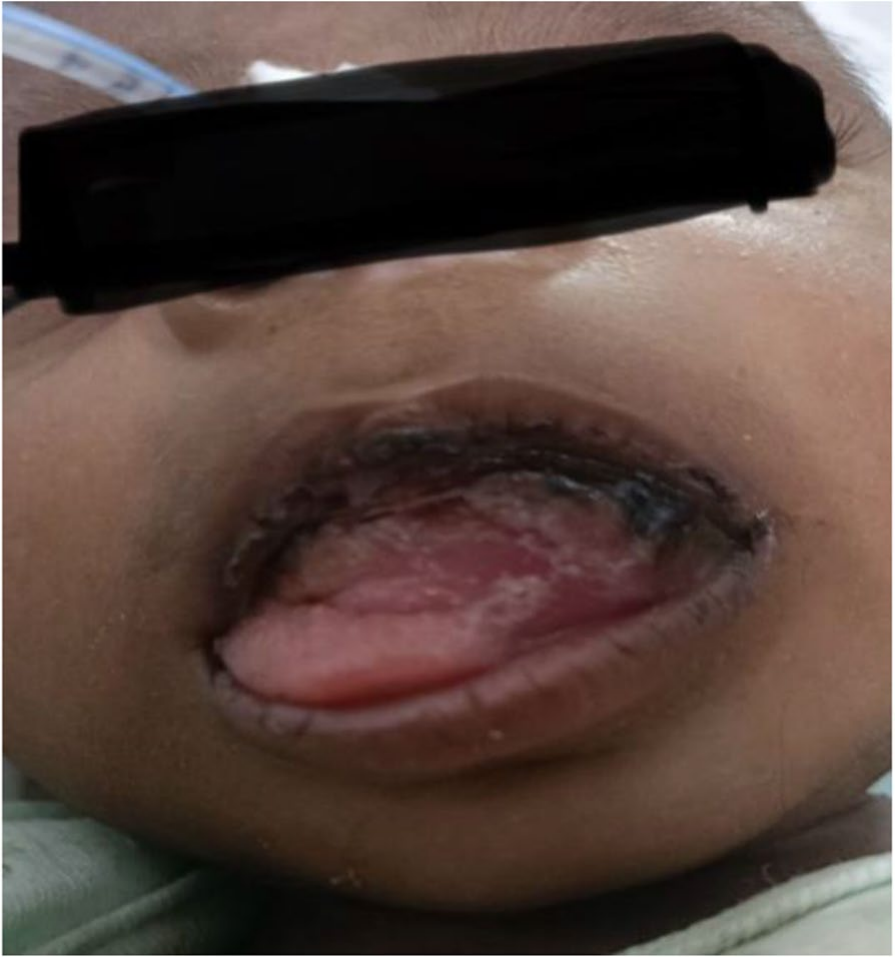
Clinical photograph obtained 3 days after embolization demonstrating marked reduction in tongue swelling with improved airway patency following successful transarterial embolization

Serial clinical follow-up was performed at 7 days, 1 month, 3 months, 6 months, and 1 year, with sustained clinical improvement and no evidence of recurrent symptoms. Follow-up MRI was planned after 3 months to evaluate for residual lesion or recurrence. No additional intervention was required during follow-up (Fig. 5).

No procedure-related complications or adverse events were observed.

## Discussion

Vascular malformations involving the tongue are uncommon congenital vascular anomalies that can result in significant morbidity in neonates because of the limited airway reserve and the functional importance of the tongue in feeding and respiration [1, 2]. Progressive enlargement or bleeding from these lesions may precipitate life-threatening airway compromise requiring urgent stabilization and intervention, as observed in the present case.

MRI and digital subtraction angiography played complementary roles in diagnosis and treatment planning. MRI accurately delineated lesion extent, intrinsic muscular involvement, and internal flow voids suggestive of a vascular malformation, while angiography enabled precise identification of the arterial supply and facilitated simultaneous endovascular treatment [3, 4].

Endovascular embolization has emerged as an important minimally invasive therapeutic option for vascular malformations of the head and neck, particularly in critically ill pediatric patients in whom surgical management may be associated with increased morbidity and intraoperative bleeding [4, 6]. However, embolization in neonates remains technically challenging because of the small caliber of vessels, increased susceptibility to vasospasm, and higher risk of vascular injury and thrombosis [5].

In the present case, vascular access was successfully achieved through the right common femoral artery using a 4-French introducer sheath, followed by superselective catheterization of the feeding lingual artery branch using a Progreat microcatheter and Neuro guidewire. Systemic heparinization and continuous heparinized saline flushing were used to minimize thrombotic complications during the procedure. Careful catheter manipulation allowed stable distal catheterization without the need for vasodilator administration.

Embolization was performed using 250 μm polyvinyl alcohol particles diluted with contrast in a 50:50 ratio. The choice of PVA particles allowed controlled distal embolization of the feeding vessels while minimizing the risk of non-target embolization. Careful technique, including slow injection and continuous fluoroscopic monitoring, was essential in achieving a safe and effective outcome. The endpoint of embolization was angiographic stasis with marked reduction in vascular blush and successful devascularization of the lesion.

Previous studies have demonstrated favorable outcomes following embolization of pediatric vascular malformations of the head and neck. Burrows et al. reported successful embolization in pediatric patients with vascular malformations, highlighting the effectiveness of endovascular techniques in controlling hemorrhage and reducing lesion vascularity [7]. Similarly, Berenstein et al. also emphasized the role of endovascular therapy in neonatal and pediatric vascular malformations with favorable clinical outcomes [5].

In our patient, embolization resulted in rapid stabilization of airway compromise, cessation of bleeding, and progressive reduction in tongue swelling without immediate procedural complications. Sustained clinical improvement was observed during serial follow-up examinations, and no additional intervention was required.

This report is limited by its single-case nature and relatively short follow-up duration. Although no recurrence was identified during follow-up, longer clinical and imaging surveillance remains necessary to evaluate for delayed recurrence, angiogenesis, or late complications following embolization.

Overall, this case highlights the important role of early imaging evaluation and specialized interventional radiology techniques in the management of neonatal tongue vascular malformations presenting with life-threatening airway compromise. Careful procedural planning, meticulous embolization technique, and structured follow-up are essential to achieve favorable and durable clinical outcomes in this vulnerable patient population.

## Conclusion

Transarterial embolization represents a safe and effective minimally invasive treatment option for neonatal tongue vascular malformations presenting with life-threatening airway compromise. In such patients, meticulous technique, including superselective catheterization and controlled delivery of appropriately sized embolic agents, is essential to achieve optimal outcomes while minimizing the risk of complications. This case highlights the importance of early diagnosis, prompt intervention, and specialized expertise in the management of vascular malformations in neonates. Careful post-procedural monitoring and planned long-term follow-up are crucial to detect potential recurrence or delayed complications. Awareness of neonatal-specific technical considerations is essential to ensure procedural safety and durable clinical outcomes.

## Data Availability

All data generated or analyzed during this study are included in this published article.
